# Productive, Reproductive, and Metabolic Responses of Replacement Gilts to Immunomodulatory Protein Concentrate Supplementation During Flushing

**DOI:** 10.3390/ani16213335

**Published:** 2026-10-23

**Authors:** Fernando Bittarello, Luciana da Silva Leal Karolewski, Gudryan Izoton, Felipe Norberto Alves Ferreira, Clarice Speridião Silva Neta, Cheila Roberta Lehnen

**Affiliations:** 1 Department of Animal Science, University State of Ponta Grossa, Ponta Grossa 84030-900, Brazil; fernandobittarello@gmail.com (F.B.); lsleal@uepg.br (L.d.S.L.K.); izotongudryan86@gmail.com (G.I.); 2 Technical Services Department, Agroceres Multimix Animal Nutrition, Rio Claro 13502-741, Brazil; felipe.alves@agroceres.com (F.N.A.F.); clarice.silva@agroceres.com (C.S.S.N.)

**Keywords:** backfat thickness, energetic diets, estrus, glucose, pig production

## Abstract

Genetic improvement, combined with the stress challenges of swine production, interferes with body condition and may impair the immunity and reproduction of gilts. The objective of this study was to estimate the productive and reproductive responses of gilts fed an immunomodulatory protein concentrate during flushing. A total of 196 gilts exhibiting second estrus were used. They were distributed in completely randomized design with two treatments (0; 40 g/d of immunomodulatory protein concentrate), distributed in blocks of light and heavy gilts. Productive and reproductive performance data, as well as metabolic parameters, were collected and subjected to analysis of variance, for which we adopted a 5% significance level. Interaction of treatment × weight × batch for average live piglet weight (*p* = 0.003) was verified. Gilts fed the diet with IPC showed no differences (*p* > 0.05) in glucose or triglyceride parameters. Flushing diets containing 40 g/d of immunomodulatory protein concentrate did not result in differences in body condition or reproductive performance of modern light and heavy gilts. Under the nutritional conditions evaluated, immunomodulatory protein concentrate supplementation therefore did not modify the metabolic profiles of gilts across weight categories.

## Simple Summary

The aim of this study was to evaluate the use of protein ingredients with immunomodulatory effects associated with an energy-rich diet during the pre-mating period in gilts. A total of 196 gilts between their second and third estrus periods were used, divided into groups of light and heavy animals, and assigned to two treatments, with or without an immunomodulatory protein concentrate. Diets containing the immunomodulatory protein concentrate did not alter the productive or reproductive responses of the gilts during flushing at farrowing.

## 1. Introduction

Modern swine production has been guided by intense genetic improvement aimed at hyperprolificacy, resulting in gilts with high growth rates and lean-tissue deposition. However, the accelerated development of modern genetic lines has increased the physiological demands associated with achieving reproductive maturity while maintaining adequate body development and nutrient reserves compared to lines from previous decades [1,2]. The period preceding first breeding represents a critical window in which nutritional management must support growth, body reserve development, and reproductive preparation without compromising the physiological condition of the female [3,4]. The metabolic balance between the demands of body growth and the maturation of the hypothalamic–pituitary–gonadal axis now constitutes the main challenge in ensuring the longevity of these females in the herd.

Moreover, the magnitude of the response to flushing may vary among gilts according to bodyweight, growth rate, body composition, and nutritional status at the beginning of the pre-breeding period [5,6]. Thus, although flushing provides a substantial nutritional stimulus before breeding, its effects may differ among females according to their initial body condition [1,5]. This variability raises the possibility that nutritional strategies combining energy and other targeted nutrients may yield additional responses beyond those obtained with the basal flushing diet alone [6,7]. Therefore, we evaluated IPC supplementation within a high-nutrient flushing program to determine whether it could further influence body condition, selected metabolic indicators, and reproductive performance.

Immunomodulatory and other functional nutritional additives have been evaluated in relation to replacement gilts, with responses varying according to the additive, feeding period, and reproductive or metabolic outcomes evaluated [8,9]. These findings indicate that the inclusion of functional nutrients may yield responses beyond those obtained from the basal diet, although such responses are not consistently observed. Therefore, evaluating IPC supplementation within a high-nutrient flushing program may determine whether an additional response can be detected under elevated-nutrient-intake conditions.

Immunomodulatory protein concentrates (IPCs) are promising tools because these additives act on endocrine signaling by optimizing immunological status, reducing the metabolic cost of inflammatory responses, and favoring nutrient partitioning for follicular development [10]. The potential benefit of incorporating IPCs into a flushing program is based on the premise that improved physiological and immune statuses may favor nutrient utilization for productive and reproductive processes during the critical pre-breeding period. Nevertheless, whether IPC supplementation provides an additional benefit beyond that achieved with flushing alone in modern replacement gilts remains unclear.

Gilts with different bodyweights may therefore respond differently to an increased nutrient supply and immunomodulatory supplementation. Evaluating these responses across bodyweight categories is important because it allows one to determine whether the IPCs’ potential benefits are consistent among modern replacement gilts or more pronounced in gilts with specific levels of body development. The objective of this study was to evaluate light and heavy replacement gilts’ productive, reproductive, and metabolic responses to immunomodulatory protein concentrate supplementation during flushing.

## 2. Materials and Methods

The use of animals for this experiment was approved by the Animal Use Ethics Committee of Agroceres Multimix (CEUA-AGMMX/Protocol No. 35.24, 26 July 2024), authorized by the Institutional Accreditation for Activities with Animals in Teaching or Research/National Council for the Control of Animal Experimentation.

### 2.1. Location, Housing, and Experimental Animals

The experiment was conducted on an experimental farm in the city of Patos de Minas/MG, Brazil. A total of 196 gilts (Agroceres PIC, Camborough, Patos de Minas, Brazil) were used, with an average live weight of 134 kg at approximately 180 to 190 days of age and at the presentation of their second estrus from August to October 2024. The daily minimum and maximum temperatures during the experimental period ranged from 14.7 ± 3.2 to 33.4 ±1.7 °C. We employed a completely randomized design with two treatments, distributed in blocks of light and heavy gilts. Each treatment included 98 replicates, with each animal considered an experimental unit. For six weeks, about 30 females/week were housed in a flushing barn. They were divided weekly into two groups according to bodyweight at the second estrus, namely, light (126.9 ± 0.442 kg) and heavy (140.8 ± 0.828 kg), and two treatment regimens, namely, Control (flushing phase diet) (Figure 1) and Control + Immunomodulatory Protein Concentrate (IPC, 40 g/d). Based on the schedule for the entry of gilts in weekly batches, following weighing, the females were randomly assigned to blinded treatments. During the flushing period, the gilts remained in individual crates equipped with nipple drinkers, trough feeders, and partially slatted concrete flooring. Exposure to a boar occurred daily in front of the individual gilt crates—in association with the back-pressure test—to identify estrus. The gilts remained in the flushing barn for approximately 21 days until identification of the third estrus, being inseminated at an average live weight of 154 kg via the cervical artificial insemination method [11].

After artificial insemination, the gilts were housed for 110 days in six collective pens in a barn with a Gestal 3G^®^ (Jyga Technologies, Quebec, QC, Canada) automated feeding system equipped with nipple drinkers, with capacity for 32 animals per pen and pen dimensions of 12.60 m × 5.18 m (2 m^2^/animal). The barn was equipped with an automatic temperature control system, featuring evaporative cooling panels with lateral exhaust fans, artificial lighting by LEDs (12 h light/12 h dark), and manure drainage gutters. After 110 days of gestation and up to the moment of farrowing, the gilts were housed in farrowing rooms, consisting of farrowing crates with a hopper feeder connected to a nipple drinker and climate control via evaporative cooling panels with lateral exhaust fans.

### 2.2. Experimental Diets and Feeding Management

The diets (Table 1) were formulated on the basis of the maintenance requirements of female pigs during the flushing and gestation periods, as per Rostagno et al.’s recommendations [12]. The corn and soybean meals used in the experimental diets were previously analyzed via near-infrared reflectance spectroscopy (NIRS) for dry matter, ash, crude fiber, ether extract, crude protein, and gross energy values.

Water and feed were provided ad libitum. The feed was supplied manually, with the schedule divided into three feedings per day (7:40 a.m., 12:30 p.m., and 3:00 p.m.), adjusted according to each gilt’s voluntary ad libitum intake. In addition to the first meal at 7:40 a.m., animals administered the IPC treatment were given 40 g/day of an additive based on amino acids (L-carnitine and L-valine), chelated minerals, vitamins (D3 and E), and plant extracts (oregano, pepper, and clove) (as presented in the footnote for Table 1), as per the manufacturer’s recommendations (Agroceres Multimix, Rio Claro, Brazil). Feeders were checked daily at each feeding, with feed leftovers removed from the feeder, dehydrated, and weighed individually. The feeders and pens were cleaned daily.

### 2.3. Evaluated Responses

#### 2.3.1. Productive Performance

Bodyweight gain and feed intake were calculated using weight measurements. During the flushing phase, gilts were weighed at the beginning of housing, on day 5, and at estrus using an individual floor scale (Trentin, model BT, Videira, Brazil). Feed intake was estimated daily via the difference between the amount offered and feed leftovers weighed on a commercial scale (Toledo do Brasil Indústria de Balanças Ltd., model 9094C/6, São Bernardo do Campo, Brazil). Average daily gain was calculated based on total weight gain from each gilt’s weighing date.

The gilts’ bodyweights, backfat thickness, deep loins, and calipers were measured in the second estrus, on the fifth day, in the third estrus, and upon entry into the farrowing house. Data on backfat thickness and loin depth were obtained using a digital ultrasonic imaging diagnosis system (Mindray, model DP-10Vet, Shenzhen, China) with a probe (Mindray, model 75L38EB; dimensions: 485 mm × 340 mm × 100 mm; frequency range: 5.0–10.0 MHz; Shenzhen, China), with the ultrasound measurements collected at region P2 (between the penultimate and last ribs, 65 mm from the dorsal midline) [13]. Caliper measurements were performed by quantifying the angulation from the spinous process to the transverse process at the last rib, as per the methodology proposed by Knauer and Baitinger [14]. Three measurements of each parameter were obtained during assessment. All measurements were performed by trained operators under blinded conditions regarding treatments.

#### 2.3.2. Reproductive Performance

The duration of the estrous cycle (the inter-estrus interval) was recorded between the second and third estrus. During the transfer of the sows to the farrowing unit, farrowing monitoring was conducted as per the farm’s standardized management practices, while the assessors remained blinded to treatment information. Reproductive performance data were collected during farrowing monitoring. Farrowing induction was not performed. Obstetric assistance during farrowing was provided in accordance with the experimental farm’s standard management protocol, with procedures documented; piglet transfers occurred between litters for cross-fostering after complete farrowing and the ingestion of colostrum by the piglets. Information on the date and time at the beginning and end of parturition was collected at the end of farrowing. The total number of piglets and the quantities of live-born, stillborn, and mummified piglets were determined via individual counting. The weights of live, stillborn, and mummified piglets, as well as the placenta, were measured with a commercial scale (Toledo do Brasil Indústria de Balanças Ltd., model 9094C/6, São Bernardo do Campo, Brazil). Average live piglet weight was calculated on the basis of the total weight and number of live piglets. The uterine content value was obtained by summing (at birth) the total number of piglets born (i.e., the litter) and placenta weights: [Uterine content (kg) = 0.3 + 1.329 × litter birth weight (kg)] (R^2^ = 0.96) [15].

#### 2.3.3. Blood Parameters

A total of 24 animals were randomly selected for blood sampling, which was performed on the 5th day of flushing and during the third estrus. The sample size was determined a priori using G*Power software (version 3.1.9.7) (Heinrich Heine University Düsseldorf, Düsseldorf, Germany) [16]. Glucose was selected as the primary outcome for sample size estimation on the basis of previous studies on gilts reporting treatment-related differences in circulating glucose concentrations (74–107 mg/dL, SD pooled = 1.07) [17,18]. A two-group F test was considered, assuming α = 0.05, 90% statistical power, and an effect size (Cohen’s f) = 0.51, resulting in a minimum of 13 animals per treatment. By puncturing the jugular vein, we collected twenty-milliliter blood samples from 24 animals (12 animals/treatment) after a two-hour fast using sterile tubes with gel and clot activator (Neo Vaccumi^®^, Kerala, India). For blood sampling, animals with bodyweights representative of the mean for each treatment and block were selected. Blood samples were collected at 10:00 a.m. after the animals had been fasting for more than two hours. Glucose and triglyceride analyses were performed using an automated biochemical analyzer (VITROS^®^ 5600 Integrated System, Ortho Clinical Diagnostics, San Diego, CA, USA) with a kit specific for glucose (VITROS Chemistry Products GLU Slides, 1707801, Ortho Clinical Diagnostics, San Diego, CA, USA) and triglyceride (VITROS Chemistry Products TRIG Slides, 1336544, Ortho Clinical Diagnostics, San Diego, CA, USA) via reflectance photometry using dry chemistry slides. All analyses were performed according to the manufacturer’s recommended protocols, and quality control procedures were conducted in accordance with the recommendations for the VITROS 5600 system (QuidelOrtho Corporation, San Diego, CA, USA).

### 2.4. Statistical Analysis

A mixed model was constructed by considering both fixed and random factors, along with their respective interactions. The fixed effects included the treatment (Control and IPC), gilt weight (light and heavy), the evaluation period (2nd estrus, 5th day at flushing, 3rd estrus, and farrowing), and all two- and three-way interactions between these structural factors. Evaluation period was modeled as a discrete categorical factor encompassing four physiological and reproductive timepoints: (2nd estrus, 5th day at flushing, 3rd estrus, and farrowing). The random effects included the housing batch (entry week), as a contemporary blocking factor to isolate seasonal environmental variations, and the individual animal (gilt) nested within the combination of batch, treatment, and weight groups. The animal was specified as the subject for repeated measures to account for temporal dependence.

The mathematical equation describing the main model is expressed as follows:

$$Y_{mijkl}=\;\mu +\;L_{m}+T_{i\;}+G_{j}\;+\left({TG}_{ij}\right)+P_{k}+\left({TP}_{ik}\right)+\left({GP}_{jk}\right)+\left({TGP}_{ijk}\right)+A_{l\left(mij\right)}+\;E_{mijkl}$$

where $Y_{mijkl}$ is the observed value of the response variable in batch m, treatment i, weight group j, period k, and animal l; μ is the overall mean; $L_{m}$ is the random effect of the m-th housing batch; $T_{i}$ is the fixed effect of the i-th treatment; $G_{j}$ is the fixed effect of the j-th initial weight group; $\left({TG}_{ij}\right)$ is the fixed interaction between the treatment and weight groups; $P_{k}$ is the fixed effect of the k-th evaluation period; $\left({TP}_{ik}\right)$ and ${(TGP}_{ijk\;})$ represent the two- and three-way fixed interactions between the factors; $A_{l(mij)}$ is the random effect of the l-th animal nested within the batch, treatment, and weight group combination; and $E_{mijkl}$ is the random residual error. The covariance structure defined for the model follows the assumptions of compound symmetry, indicating that the collected periods exhibit homogeneous variance.

Prior to model validation and hypothesis testing, residuals were subjected to rigorous diagnostic checks to ensure compliance with general linear-model assumptions, and values with residuals >3 or <−3 were removed as outliers. A high number of stillborn and mummified piglets from two gilts removed after farrowing were considered outliers. Residual normality was verified using the Anderson–Darling test coupled with visual inspection of normal probability plots (Q–Q plots). The normality of the random-effect estimates (BLUPs) for individual gilts and batches was assessed using quantile–quantile (Q–Q) plots. Residuals were subjected to Levene’s test for homoscedasticity. Subsequently, the overall data were subjected to the Anderson–Darling normality test (*p*-value > 0.05).

Following mixed-model verification, evaluation period (2nd estrus, 5th day at flushing, 3rd estrus, and farrowing) was excluded from the main model whenever the mean values for this effect were reported by period in the tables. Global *p*-values for all main fixed effects and interactions were fully maintained and are reported for each variable in the tables. However, the partitioning (slicing) of means for three-way interactions (treatment × group × period) was omitted from the tables if the global interaction effect was non-significant (*p* > 0.05). In such scenarios, these specific combinations were removed from the mean tables, and only the main effects or two-way interactions that remained statistically significant were reported in detail. Least-squares means (LS-means) and standard deviations are presented in the tables. When the main effects or interactions of the fixed effects (treatment and weight) were statistically significant in the analysis of variance (*p* < 0.05), least-squares means (LS-means) were compared using Tukey’s test.

Farrowing success (binary outcome: 0 = failure and 1 = success) was analyzed using a generalized linear mixed-effects model with a binomial distribution and a logit link function. Treatment, weight category (light or heavy), and their interaction were included as fixed effects, whereas batch was included as a random intercept to account for between-batch variability. Model parameters were estimated by maximum likelihood using the Laplace approximation, as implemented in the glmer function of the lme4 package. Estimated marginal means were obtained using the emmeans package, back-transformed to the response scale, and expressed as percentages. Pairwise comparisons among treatment groups were performed using model-based contrasts, with *p*-values adjusted using the Holm procedure to control the family-wise error rate. Statistical significance was set at *p* < 0.05. Model convergence and parameter stability were assessed before interpretation of the results. All analyses were performed using R software (version 4.6.1; R Core Team, Vienna, Austria) [19]. The proportion of stillborn and mummified piglets relative to the total number born—previously adjusted using the arcsine square root transformation—was analyzed using a linear mixed model, considering the fixed effects of treatment and weight and the random effect of batches. Pearson correlation coefficients, along with their 95% confidence intervals, were calculated to evaluate linear relationships between productive performance and metabolic parameters. Multicollinearity among variables was disregarded. To control the Type I error inflation arising from the multiple simultaneous hypothesis tests, the Benjamini–Hochberg False-Discovery Rate (FDR) procedure was applied. All correlation tests performed in this study were considered a single family of tests. Correlations were considered statistically significant at FDR-adjusted *p* < 0.05.

Covariates were predefined using the backward elimination method, with only those showing statistical significance (*p* < 0.05) retained in the model. Inter-estrus interval was used as a covariate in metabolic parameters. Missing data bias was controlled using the REML method for mixed models for each variable. All statistical analyses were performed using Minitab Statistical Software (version 22.1; Minitab, LLC, State College, PA, USA) [20].

## 3. Results

### 3.1. Productive Performance

The gilts’ productive performance assessed on the basis of body condition (BW, BFT, LD, and caliper) and flushing duration did not differ (*p* > 0.05) between the IPC and Control groups (Table 2). Between light and heavy gilts, it was possible to clearly distinguish differences in bodyweight (*p* < 0.001), BFT and LD, and caliper values (*p* < 0.001), but there were no differences (*p* > 0.05) during the flushing period. As expected, the body condition of gilts at second estrus, on the fifth day of flushing, and during third estrus differed over time (*p* < 0.001). Growth rates for weight gain, backfat, and loin were proportional between light and heavy gilts. However, the differences between estrus (second to third) regarding weight gain, backfat thickness, and loin depth did not differ between treatments and groups. Furthermore, the gilts gained bodyweight throughout the flushing period up to near farrowing. However, IPC supplementation during flushing did not lead to differences (*p* > 0.05) in bodyweight, BFT, or LD in the gilts in the period evaluated. The batches did not differ (*p* > 0.05) from one another, nor did they differ regarding the treatments and groups of light and heavy gilts. Table 3 presents a global analysis, with most effects evaluated in light and heavy gilts fed diets containing immunomodulatory protein concentrate during the flushing period. Light and heavy gilts differed (*p* < 0.05) regarding BW, BFT, LD, and caliper measurements. The periods evaluated—second estrus, fifth day of flushing, third estrus, and farrowing—differed (*p* < 0.05) from one another regarding gilt body performance from the second estrus through farrowing. Dietary IPC and weekly batches did not differ (*p* > 0.05) regarding the evaluated parameters. Treatment × weight, treatment × period, treatment × weight × batch, and treatment × weight × period interactions showed no significant (*p* > 0.05) differences for the variables evaluated (BW, BFT, LD, and caliper).

Daily feed intake and cumulative feed intake during flushing did not differ (*p* > 0.05) between treatments (Table 4). Daily average and cumulative feed intake were similar (*p* > 0.05) between light and heavy gilts. Daily weight gain did not differ (*p* > 0.05) between treatments and groups.

### 3.2. Reproductive Performance

Gilts that showed reproductive and locomotor problems preventing proper experimental development and hindering animal welfare during the gestation period were removed from the study. In total, 14 and 7 females were removed from the control and IPC treatments, respectively. These 21 gilts were removed because of prolonged anestrus during the third estrus, vulvar discharge shortly after service, lameness and abortion during gestation, and complete piglet death at farrowing, as shown in Table 5.

Gilts at parturition that were fed diets with or without IPC during flushing showed no differences (*p* > 0.05) in body condition or reproductive performance (Table 6). Bodyweight, loin depth, and caliper at farrowing differed (*p* < 0.05) between gilts from the light and heavy groups, with no differences (*p* > 0.05) in weight gain or backfat thickness during gestation. A significant interaction of treatment × weight × batch for average live piglet weight (*p* = 0.003) was verified. In this interaction, all means were similar, except for the higher piglet birth weight from heavy gilts fed diets containing IPC (from batch 6, B6) in relation to the average live piglet weight of light gilts from the control group (from batch 1, B1). For the remaining variables, no significant difference or interaction was observed.

### 3.3. Metabolic Parameters

The glucose and triglyceride responses show that the use of the immunomodulatory protein concentrate associated with the flushing diet did not differ on the fifth day of flushing and at estrus (Table 7). After the Benjamini–Hochberg FDR method was applied, correlations between performance and metabolic parameters (glucose and triglycerides) on the fifth day of flushing and at estrus were not significant (*p* > 0.05) (results are not presented).

## 4. Discussion

As expected, light and heavy gilts differed significantly in bodyweight, backfat thickness, caliper measurements, and loin depth, confirming that the classification according to initial body size effectively differentiated the animals according to their body development and tissue reserves. Growth and body development during the pubertal period play a central role in determining gilts’ physiological readiness for entry into the breeding herd. This response was consistent across weight categories, as no treatment × weight-category interactions were detected. Thus, although light and heavy gilts differed in their initial body reserves, there was no statistical evidence that these differences modified the response to the immunomodulatory treatment under the nutritional conditions in this study.

Adequate accumulation of lean tissue and subcutaneous fat reserves, reflected by bodyweight and backfat thickness, is essential for achieving an appropriate body condition at first service and supporting subsequent reproductive performance [21,22]. Because the optimal body condition varies with genotype and production system, first-service targets should be defined within biologically appropriate ranges rather than based on bodyweight alone. Achieving these targets may help improve reproductive efficiency, herd retention, and productive longevity [22,23]. Although the gilts were allocated to groups, all animals presented body conditions within the expected reproductive range, achieving target live weights, backfat thickness (BFT), and dorsal fat thickness of 135–160 kg, 15–20 mm, and 13–15 mm, respectively, as proposed in other studies [1,5,24]. In commercial herds, monitoring the body condition—specifically backfat thickness and loin depth— of hype prolific sows is essential, particularly because gilts accumulate less fat during development leading up to farrowing and exhibit greater loin loss after first parity than multiparous sows [25].

Gilts with a live weight exceeding 160 kg at breeding exhibit impaired reproductive performance, reduced oocyte quality, and smaller litter sizes, with an increased risk of second-parity syndrome [26]. Excessive bodyweight also increases the incidence of lameness, thereby compromising sow welfare and piglet development, shortening productive longevity, and raising culling rates during early parities [24,27]. Gilt weight at the onset of the productive phase is determined by multiple factors that influence body deposition; consequently, weight gain during this period is critical for optimal reproductive performance, longevity, and retention in the herd [28]. Body condition reflects both genetic selection and nutritional management during growth [29], and individual variation in growth trajectories under ad libitum feeding likely accounts for the differences observed in live weight and backfat thickness at study entry. Despite their greater lean reserves at the onset of gestation, gilts’ progressive development of fetal, placental, uterine, and mammary tissues increases nutrient and amino acid requirements throughout pregnancy [30]. This relationship may be particularly relevant in modern hyperprolific females, for which protein and amino acid requirements increase substantially during late gestation [31]. Moreover, energy and protein supply influence nitrogen retention and the reconstitution of maternal body reserves during gestation [31,32].

Feed consumption met the daily growth nutritional requirements for gilts of modern genetic lines. Considering this nutritional adjustment, the feed ratio regarding gain for gilts fed a diet with or without IPC during flushing was adjusted to the growth curves for each female. This response may reflect greater loin accretion when nutrient supply is aligned with the growth demands of gilts with lower initial body reserves. In this study, the flushing program provided a good nutritional stimulus to the gilts, which consumed approximately 4.1 kg/d of a diet containing 3291 kcal of metabolizable energy/kg for approximately 21 days.

A total of 21 gilts were removed during the experimental period, with seven removals in each of the light control, heavy control, and heavy IPC groups, and none in the light IPC group. The recorded reasons included reproductive conditions (anestrus, abortion, vulvar discharge, and an unfavorable farrowing outcome) and health- or behavior-related events (lameness, death, failure to eat, and stress-related behavior). These events occurred at different stages of the experimental period and may reflect health- and management-related challenges under the conditions of the commercial farm. Nevertheless, the absence of removals in the light IPC group, compared with seven removals in the corresponding light control group, represents an imbalance that warrants consideration when interpreting the reproductive outcomes. Although the reasons for removal were documented, a potential association between treatment allocation and the occurrence of these events cannot be definitively ruled out. Therefore, the differences observed in farrowing success should be interpreted with caution, and a direct causal effect of IPC supplementation should not be inferred without further supporting evidence.

Although circulating glucose and triglyceride concentrations did not differ significantly between treatments on day 5 of flushing or at estrus, their associations with feed intake and body condition indicate that metabolic variation was more closely related to individual nutritional status than to IPC supplementation. This interpretation is consistent with the similar metabolic responses observed between treatment groups despite differences in initial bodyweight and body reserves [33]. Therefore, under the high nutrient intake provided during flushing, individual variation in feed intake and body composition may have contributed more to circulating metabolic concentrations than the additional inclusion of IPC.

The lack of significant effects of IPC may indicate that the nutritional conditions provided during flushing limited the potential for detecting an additional metabolic response to immunomodulation. Because the diet supplied a high level of energy and nutrients and supported the expected growth of the gilts, the physiological response associated with nutritional stimulation may have predominated over any additional effect attributable to IPC. Studies show that providing energy-dense diets in the pre-breeding period improves reproductive outcomes in gilts by increasing ovulation rates and embryo survival [5,6,24]. Previous studies suggest that increases in nutrient availability before breeding can influence reproductive function through metabolic and neuroendocrine pathways [5,25]. Furthermore, absorption of nutrients from a diet enables appropriate live-weight and BFT gain for reproduction, leading to better body condition for the gestation period, thus impacting gilt longevity [6].

Overall, IPC supplementation did not have significant additional effects on body development and reproductive performance responses under the nutritional conditions imposed in this study. These results differ from those found in the literature, where a physiological contribution to gilt homeostasis and an increase in the embryonic survival rate may occur [2,11,26]. Although light and heavy gilts differed in initial body reserves, both groups maintained comparable patterns of body development during flushing. These findings suggest that the nutritional management strategy adopted during the pre-breeding period was sufficient to support the development of gilts across different initial weight categories, potentially limiting the magnitude of any additional responses to IPC. Furthermore, they indicate that a 40 g/day dose of the immunomodulator does not influence metabolic and reproductive responses.

## 5. Conclusions

Flushing diets containing 40 g/d of immunomodulatory protein concentrate did not result in differences in body condition or reproductive performance in modern light and heavy gilts. Under the nutritional conditions evaluated, immunomodulatory protein concentrate supplementation therefore did not modify the metabolic profiles of gilts across weight categories.

## Figures and Tables

**Figure 1:**
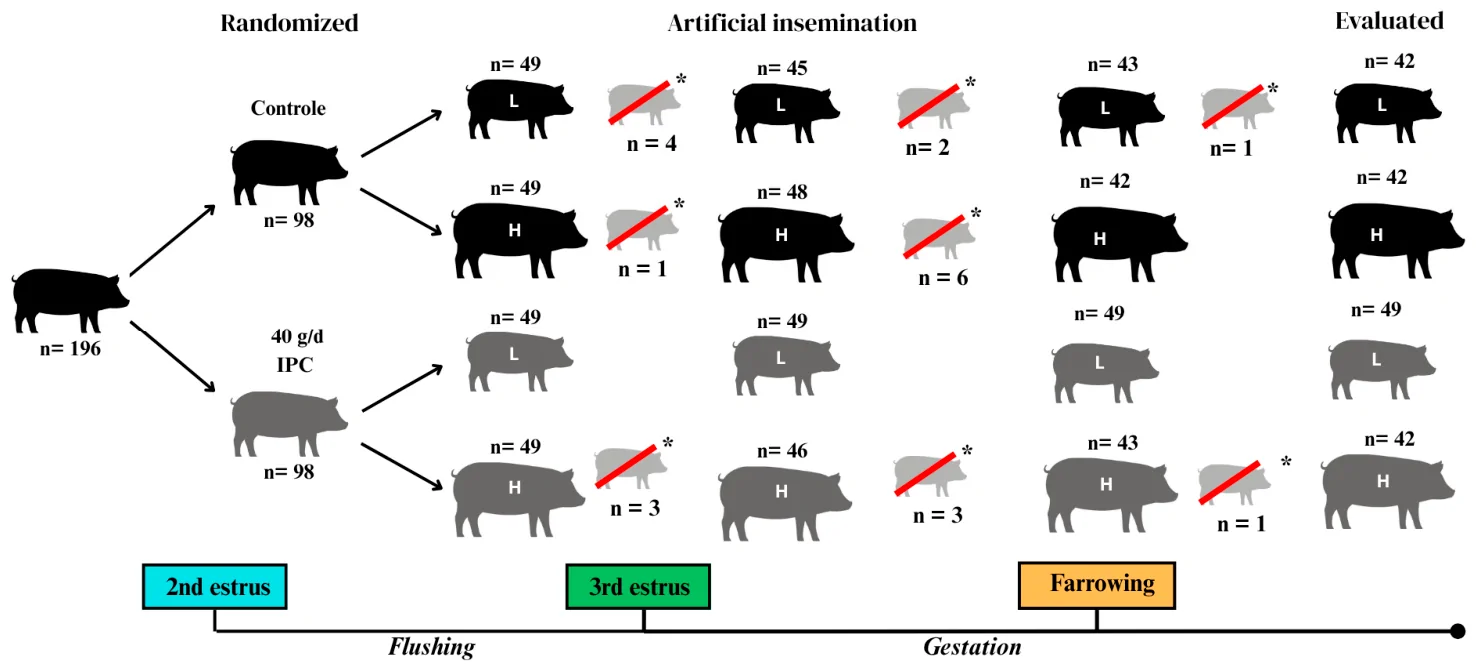
Schematic view of the experimental design. A total of 196 replacement gilts in their second estrus were selected for the study and randomly assigned to two treatments (Control or 40 g/d of IPC) during the flushing phase. Of the 98 females selected in each treatment, 49 were grouped into light or heavy classes (L and H, respectively); they were administered the treatment until insemination on the third estrus. * Discarding gilts for health or reproductive issues.

**Table 1:** Composition and nutrient levels of the basal diet of gilts during the flushing and gestation phases.

Item		Diets	
Ingredients	Unit	Flushing	Gestation
Corn	kg	714.84	680.38
Soybean meal 46% CP	kg	95.00	187.12
Biscuit meal	kg	100.0	-
Wheat bran	kg	-	100.0
Sugar	kg	50.00	-
Calcitic limestone	kg	9.82	9.75
Dicalcium phosphate	kg	10.17	10.42
Common salt	kg	5.61	5.01
L-Lysine Monohydrochloride 99%	kg	3.13	-
DL-Methionine 99%	kg	1.23	-
L-Threonine 98.5%	kg	1.54	-
L-Tryptophan 60%	kg	0.38	-
L-Valine 98%	kg	0.27	-
Premix ^1^	kg	5.00	5.00
Adsorbent ^2^	kg	3.00	2.50
Total	kg	1000	1000
Immunomodulatory Protein Concentrate ^3^	g/d	40.00	-
Nutrients			
Dry matter	%	88.97	87.78
Mineral matter	%	4.630	5.360
Crude protein	%	11.16	15.03
Ether Extract	%	3.640	2.920
Starch	%	46.03	45.20
Crude fiber	%	1.990	3.020
Acid Detergent Fiber	%	2.560	4.190
Neutral Detergent Fiber	%	9.520	13.30
Total Calcium	%	0.840	0.850
Total Phosphorus	%	0.440	0.550
Available Phosphorus	%	0.390	0.410
Phytase	FTU/kg	600	510
Sodium	%	0.240	0.220
Metabolizable Energy	kcal/kg	3291	3100
Digestible Lysine	%	0.640	0.650
Digestible Methionine + Cystine	%	0.460	0.440
Digestible Threonine	%	0.480	0.470
Digestible Tryptophan	%	0.120	0.160
Digestible Valine	%	0.490	0.650

^1^ Mineral and vitamin premix supplied by Agroceres Multimix (Rio Claro, São Paulo, Brazil). Supply per kg of diet (as-fed basis): Calcium (min) 205.33 g (max) 227.33 g, Copper (min) 3465.00 mg, Chromium (min) 6.66 mg, Iron (min) 4000.00 mg, Fluorine (max) 650.00 mg, Phosphorus (min) 65.00 g, Iodine (min) 60.00 mg, Manganese (min) 2400.00 mg, Selenium (min) 11.67 mg, Sodium (min) 70.00 g, Zinc (min) 4800.00 mg, Folic Acid (min) 50.00 mg, Pantothenic Acid (min) 666.70 mg, Biotin (min) 16.70 mg, Choline (min) 20.00 g, Niacin (min) 1166.90 mg, Vitamin A (min) 333,400.00 IU, Vitamin B1 (min) 73.30 mg, Vitamin B12 (min) 833.45 mcg, Vitamin B2 (min) 133.30 mg, Vitamin B6 (min) 73.30 mg, Vitamin D3 (min) 112,520.00 IU, Vitamin E (min) 1667.00 IU, Vitamin K3 (min) 110.00 mg, Phytase (min) 16,670.00 FT. ^2^ Mycotoxin adsorbent additive supplied by GRASP (Curitiba, Paraná, Brazil). Supply per kg of diet (as-fed basis): Bentonite 690 g; Yeast cell wall 300 g; Silymarin ≥ 500 mg. ^3^ Immunomodulatory protein concentrate supplied by Agroceres Multimix (Rio Claro, São Paulo, Brazil). Amount per kg of product: Crude Protein (min) 100.00 g, Ether Extract (min) 20.00 g, Crude Fiber (max) 200.00 g, Ash (max) 100.00 g, Moisture (max) 130.00 g, Carnitine (min) 1000.00 mg, Lysine (min) 5000.00 mg, Threonine (min) 3000.00 mg, Tryptophan (min) 1000.00 mg, Valine (min) 10.00 g, Calcium (min) 450.00 mg (max) 9350.00 mg, Chromium (min) 5.00 mg, Phosphorus (min) 1000.00 mg, Selenium (min) 5.00 mg, Sodium (min) 1500.00 mg, Zinc (min) 100.00 mg, Vitamin A (min) 50,000.00 IU, Vitamin D3 (min) 25,000.00 IU, Vitamin E (min) 30.00 IU, *Bacillus licheniformis* 16,000.00 × 10^6^ CFU, *Bacillus subtilis* 16,000.00 × 10^6^ CFU, DHA (min) 7000.00 mg. Provided per 40 g daily dose (recommended by the manufacturer when supplied on top): Crude Protein (min) 4.00 g, Ether Extract (min) 0.80 g, Crude Fiber (max) 8.00 g, Ash (max) 4.00 g, Moisture (max) 5.20 g, Carnitine (min) 40.00 mg, Lysine (min) 200.00 mg, Threonine (min) 120.00 mg, Tryptophan (min) 40.00 mg, Valine (min) 0.40 g, Calcium (min) 18.00 mg (max) 374.00 mg, Chromium (min) 0.20 mg, Phosphorus (min) 40.00 mg, Selenium (min) 0.20 mg, Sodium (min) 60.00 mg, Zinc (min) 4.00 mg, Vitamin A (min) 2000.00 IU, Vitamin D3 (min) 1000.00 IU, Vitamin E (min) 1.20 IU, *Bacillus licheniformis* 640.00 × 10^6^ CFU, *Bacillus subtilis* 640.00 × 10^6^ CFU, DHA (min) 280.00 mg.

**Table 2:** Performance of light and heavy gilts fed diets containing or not containing the immunomodulatory protein concentrate during the flushing period.

	Control		IPC		IC 95%			Effect				
	Light ^1^	Heavy ^2^	Light ^1^	Heavy ^2^	Treat	Weight	RSD	T	W	T*W	B	T*W*B
N gilts	49	49	49	49								
BW at 2nd estrus, kg	126.9 ^b^	140.8 ^a^	126.9 ^b^	140.8 ^a^	(−1.760; 1.816)	(12.14; 15.71)	6.486	0.930	<0.001	0.983	0.102	0.991
BW 5th day, kg	135.0 ^b^	147.2 ^a^	133.8 ^b^	147.8 ^a^	(−2.141; 1.679)	(11.22; 15.04)	6.977	0.528	<0.001	0.150	0.113	0.923
BW at 3rd estrus, kg	147.0 ^b^	160.0 ^a^	146.6 ^b^	160.1 ^a^	(−2.490; 2.220)	(10.95; 15.64)	8.393	0.801	<0.001	0.766	0.206	0.776
Weight gain 2nd–3rd, kg	19.99	19.14	19.63	19.36	(−1.900; 1.749)	(−2.344;1.305)	6.539	0.908	0.262	0.743	0.206	0.504
BFT at 2nd estrus, mm	12.06 ^b^	13.29 ^a^	11.84 ^b^	14.15 ^a^	(−0.530; 1.186)	(0.912; 2.628)	3.046	0.574	0.002	0.325	0.501	0.804
BFT 5th day, mm	12.47 ^b^	13.83 ^a^	12.56 ^b^	14.38 ^a^	(−0.523; 1.167)	(0.748; 2.438)	3.066	0.408	<0.001	0.372	0.118	0.934
BFT at 3rd estrus, mm	14.63 ^b^	16.05 ^a^	14.88 ^b^	16.28 ^a^	(−0.735; 1.215)	(0.431; 2.382)	3.466	0.468	0.014	0.992	0.105	0.337
BFT gain 2nd-3rd, mm	2.640	2.746	3.020	2.062	(−1.900; 1.749)	(−2.344; 1.305)	2.198	0.694	0.214	0.106	0.087	0.303
LD at 2nd estrus, mm	50.59 ^b^	54.79 ^a^	51.91 ^b^	54.09 ^a^	(−0.939; 1.506)	(1.949; 4.394)	4.337	0.657	0.003	0.105	0.985	0.200
LD 5th day, mm	50.71 ^b^	53.78 ^a^	51.38 ^b^	53.33 ^a^	(−1.090; 1.291)	(1.323; 3.704)	4.169	0.877	0.025	0.607	0.494	0.170
LD at 3rd estrus, mm	54.32 ^b^	56.08 ^a^	54.25 ^b^	56.55 ^a^	(−1.363; 1.705)	(0.458; 3.526)	5.355	0.851	0.039	0.734	0.768	0.191
LD gain 2nd–3rd, mm	3.792	1.315	2.337	2.523	(−1.566; 1.315)	(−2.620; 0.261)	5.009	0.908	0.123	0.130	0.469	0.366
Caliper at 2nd, mm	10.21 ^b^	11.77 ^a^	10.78 ^b^	11.87 ^a^	(−0.067; 0.715)	(0.941; 1.724)	1.400	0.136	<0.001	0.254	0.144	0.568
Caliper 5th day, mm	10.88 ^b^	12.18 ^a^	11.14 ^b^	12.21 ^a^	(−0.231; 0.494)	(0.835; 1.559)	1.317	0.320	<0.001	0.471	0.747	0.717
Caliper at 3rd, mm	12.41 ^b^	13.62 ^a^	12.77 ^b^	13.70 ^a^	(−0.225; 0.662)	(0.632; 1.519)	1.599	0.072	0.002	0.180	0.022 *	0.964
Caliper 2nd–3rd, mm	2.192	1.901	1.999	1.821	(−0.557; 0.294)	(−0.661; 0.190)	1.529	0.520	0.279	0.793	0.003 **	0.912
Flushing days	20.59	20.92	20.36	20.35	(−1.376; 0.599)	(−0.829; 1.147)	3.481	0.432	0.604	0.873	0.541	0.250

IPC: Immunomodulatory protein concentrate. Treat (T): Treatment-Control/IPC. Weight (W): ^1^ Light-weight range = 119–137 kg. ^2^ Heavy-weight range = 137–170 kg. Batches (B). Interactions between factors. T*W; T*W*B. ^a,b^ LS-Means in the same row followed by different lowercase letters differ according to Tukey’s test (*p* < 0.05). RSD: Residual standard deviation. BFT: Backfat thickness. LD: Loin depth. * Caliper at 3rd, mm (LS-Means for batches) = 1(12.41); 2(12.93); 3(12.35); 4(13.54); 5(13.54); 6(14.1); ** Caliper 2nd–3rd, mm (LS-Means for batches) = 1(0.24); 2(1.53); 3(1.51); 4(2.52); 5(2.74); 6(3.64).

**Table 3:** Treatment, weight, and period evaluated on performance of light and heavy gilts fed diets containing or not containing the immunomodulatory protein concentrate during flushing.

	Treat		Group		Period					Effect							
	Control	IPC	Light ^1^	Heavy ^2^	2nd e	5th d	3rd e	Farrowing	RSD	T	W	T*W	B	T*W*B	*p*	T*P	T*W*P
BW, kg	164.17	163.8	157.1 ^b^	170.9 ^a^	133.9 ^d^	141.0 ^c^	153.5 ^b^	227.6 ^a^	10.192	0.753	0.001	0.450	0.075	0.505	<0.001	0.980	0.211
BFT, mm	14.24	14.42	13.57 ^b^	15.08 ^a^	12.82 ^b^	13.30 ^b^	15.44 ^a^	15.74 ^a^	3.320	0.672	0.002	0.718	0.572	0.230	<0.001	0.884	0.831
LD, mm	52.76	52.97	51.66 ^b^	54,07 ^a^	52.86 ^b^	52.32 ^b^	55.32 ^a^	50.95 ^c^	4.909	0.568	0.014	0.514	0.933	0.519	<0.001	0.996	0.519
Caliper, mm	12.11	12.32	11.70 ^b^	12.73 ^a^	11.15 ^b^	11.60 ^b^	13.12 ^a^	12.98 ^a^	1.591	0.066	<0.001	0.064	0.206	0.504	<0.001	0.934	0.885
	Interval Confidence 95%				2nd–Farr	5th d–Farr	5th d–3rd e										
BW, kg	(−1.760; 1.816)		(12.14; 15.71)		(−31.30; −28.81)	(−24,24; −21.76)	(−11.79; −9.285)										
BFT, mm	(−0.328; 0.149)		(−0.996; −0.520)		(−1.914; −1.102)	(−1.430; −0.616)	(0.704; 1.522)										
LD, mm	(−0.455; 0.249)		(−1.556; −0.852)		(−0.604; 0.596)	(−1.148; 0.052)	(1.856; 3.065)										
Caliper, mm	(−0.218; 0.007)		(−0.628; −0.403)		(−1.251; −0.866)	(−0.807; −0.422)	(0.712; 1.099)										

IPC: Immunomodulatory protein concentrate. Treat (T): Treatment-Control/IPC. Weight (W): ^1^ Light-weight range= 119–137 kg. ^2^ Heavy-weight range = 137–170 kg. Batches (B). Period (P): 2nd estrus, 5th day at flushing, 3rd estrus, farrowing. Interactions between factors. T*W; T*W*B; T*P T*W*P. ^a,b,c^ Means in the same row followed by different lowercase letters differ according to Tukey’s test (*p* < 0.05). RSD: Residual standard deviation. BFT: Backfat thickness. LD: Loin depth.

**Table 4:** Feed intake and weight gain of light and heavy gilts fed diets containing or not containing immunomodulatory protein concentrate during the flushing period.

	Control		IPC			Effect		
	Light ^1^	Heavy ^2^	Light ^1^	Heavy ^2^	RSD	Treat	Weight	T*W
N gilts	49	49	49	49				
ADFI, kg/d^3^	4.074	4.087	4.042	4.137	0.429	0.879	0.380	0.507
TCFI, kg	76.77	77.21	75.75	77.89	11.50	0.918	0.433	0.605
ADG, kg/d	0.987	0.911	0.965	0.950	0.274	0.828	0.244	0.411

IPC: Immunomodulatory protein concentrate. Treat (T): Treatment-Control/IPC. ^1^ Weight (W): Light-weight range = 119–137 kg. ^2^ Heavy-weight range = 137–170 kg. Interactions between factors T*W. LS-Means in the same row followed by different lowercase letters differed according to Tukey’s test (*p* < 0.05). ADFI: Average daily feed intake. TCFI: Cumulative feed intake until estrus. ADG: Average daily gain. RSD: Residual standard deviation.

**Table 5:** Reasons for culling or discarding gilts fed diets containing or not containing the immunomodulatory protein concentrate during flushing up to farrowing.

ID Gilt	Treatment	Weight	Removal Timing	Major Reason	Specific Causes
01	Control	Light	3rd week	Reproductive	Anestrus
02	Control	Light	3rd week	Health	Behavior, did not eat
03	Control	Light	3rd week	Health	Lameness
04	Control	Light	3rd week	Health	Death
05	Control	Light	Gestation	Health	Vulvar discharge
06	Control	Light	Gestation	Health	Lameness
07	Control	Light	Farrowing	Reproductive	All piglets stillborn
08	Control	Heavy	3rd week	Reproductive	Anestrus
09	Control	Heavy	Gestation	Reproductive	Vulvar discharge
10	Control	Heavy	Gestation	Health	Death
11	Control	Heavy	Gestation	Health	Lameness
12	Control	Heavy	Gestation	Health	Vulvar discharge
13	Control	Heavy	Gestation	Health	Lameness
14	Control	Heavy	Gestation	Reproductive	Abortion
15	IPC	Heavy	3rd week	Reproductive	Anestrus
16	IPC	Heavy	3rd week	Reproductive	Anestrus
17	IPC	Heavy	3rd week	Health	Death
18	IPC	Heavy	Gestation	Health	Vulvar discharge
19	IPC	Heavy	Gestation	Health	Lameness
20	IPC	Heavy	Gestation	Health	Behavior, stress
21	IPC	Heavy	Farrowing	Reproductive	Vulvar discharge, all piglets stillborn

IPC: Immunomodulatory protein concentrate.

**Table 6:** Productive and reproductive performance of light and heavy gilts fed, at farrowing, diets containing or not containing the immunomodulatory protein concentrate during the flushing period.

	Control		IPC		IC 95%			Effect				
	Light ^1^	Heavy ^2^	Light ^1^	Heavy ^2^	Treat	Weight	RSD	T	W	T*W	B	T*W*B
N gilts at farrowing	42	42	49	42								
Farrowing weight, kg	219.3 ^b^	237.0 ^a^	221.5 ^b^	232.7 ^a^	(−6.17; 4.06)	(9.29; 19.52)	16.81	0.907	<0.001	0.236	0.398	0.427
BW gain during pregnancy, kg	71.87	77.03	74.94	72.86	(−5.16; 4.06)	(−3.07; 6.15)	15.41	0.968	0.309	0.123	0.893	0.226
BFT at farrowing, mm	15.21 ^b^	16.45 ^a^	15.10 ^b^	16.31 ^a^	(−1.338; 1.086)	(0.009; 2.433)	4.069	0.865	0.012	0.965	0.201	0.303
LD at farrowing, mm	49.27 ^b^	52.20 ^a^	50.69 ^b^	51.23 ^a^	(−1.343; 2.086)	(0.167; 3.596)	5.764	0.582	0.104	0.237	0.767	0.432
Caliper at farrowing, mm	12.30 ^b^	13.32 ^a^	12.99 ^b^	13.13 ^a^	(−0.392; 0.722)	(−0.046; 1.068)	1.851	0.620	0.082	0.127	0.614	0.682
Gestation days, d	114.9	115.1	115.1	115.4	(−0.120; 0.533)	(−0.079; 0.574)	1.105	0.187	0.062	0.946	0.433	0.649
Farrowing rate, %	81.6	87.7	100	85.7	(−0.060; 1.694)	(−1.285; 0.469)	0.485	0.652	0.277	0.526	0.230	0.620
Total piglets, *n*	17.10	16.32	16.44	16.47	(−1.344; 0.535)	(−1.284; 0.595)	3.011	0.140	0.674	0.384	0.695	0.120
Live-born piglets, *n*	16.12	14.92	15.14	15.36	(−1.180; 0.630)	(−1.398; 0.412)	2.922	0.277	0.545	0.125	0.797	0.239
Live-born piglets’ weight, kg	21.13	20.62	20.35	20.89	(−1.530; 1.026)	(−1.265; 1.291)	4.246	0.743	0.854	0.576	0.020	0.070
Average live piglets’ weight, kg	1.371	1.414	1.347	1.369	(−0.096; 0.0275)	(−0.029; 0.094)	0.191	0.363	0.690	0.969	0.987	0.003 **
Stillborn, %	0.478	0.513	0.444	0.390	(−0.357; 0.217)	(−0.297; 0.277)	0.960	0.679	0.963	0.761	0.661	0.508
Mummified, %	0.324	0.459	0.575	0.320	(−0.269; 0.389)	(−0.386; 0.272)	1.078	0.598	0.639	0.252	0.593	0.335
Placental weight, kg	3.562	2.979	3.396	3.409	(−0.235; 0.505)	(−0.660; 0.081)	1.240	0.371	0.066	0.111	0.752	0.395
Uterine contents, kg *	25.08	23.86	24.68	25.04	(−1.865; 1.118)	(−1.650; 1.332)	5.044	0.345	0.619	0.356	0.314	0.119
Farrowing duration, min	256.5	260.8	254.3	241.8	(−41.9; 3.50)	(−25.1; 20.3)	75,57	0.063	0.880	0.463	0.340	0.916

IPC: Immunomodulatory protein concentrate. Treat (T): Treatment-Control/IPC. RSD: Residual standard deviation. BW: Bodyweight. BFT: Backfat thickness. LD: Loin depth. ^1^ Weight (W): Light-weight range = 119–137 kg; ^2^ heavy-weight range = 137–170 kg * Estimated by [15]. Means in the same row followed by different lowercase letters differ according to Tukey’s test (*p* < 0.05). ** Average live piglets’ weight, kg (LS-Means for Treatment*Weight*Batches) = IPC*heavy*B6 (1.589 ^a^); Control*heavy*B2 (1.539 ^ab^); Control*light*B4 (1.536 ^ab^); IPC*heavy*B5 (1.472 ^ab^); IPC*heavy*B4(1.448 ^ab^); IPC*heavy*B1 (1.438 ^ab^); IPC*light*B4 (1.427 ^ab^); IPC*light*B6 (1.415 ^ab^); Control* heavy*B3 (1.391 ^ab^); Control*light*B3 (1.384 ^ab^); Control*heavy*B4 (1.362 ^ab^); IPC*light*B3(1.358 ^ab^); IPC*light*B2 (1.354 ^ab^); IPC*heavy*B3 (1.353 ^ab^); Control*heavy*B5 (1.336 ^ab^); Control*light*B5 (1.330 ^ab^); Control*light*B6 (1.324 ^ab^); IPC*light*B5 (1.318 ^ab^); IPC*heavy*B2 (1.316 ^ab^); Control*heavy*B4 (1.314 ^ab^); IPC*light*B1 (1.310 ^ab^); Control*light*B2 (1.279 ^ab^); Control*heavy*B4 (1.186 ^ab^); Control*light*B1 (1.157 ^b^).^a,b^ Different superscript letters for (LS-Means interactions) differ by Tukey test (*p* < 0.05).

**Table 7:** Metabolic parameters of gilts fed diets containing or not containing the immunomodulatory protein concentrate during flushing and estrus.

	Glucose, mg/dL *									
Period	Control	IPC	IC95%	RSD	T	W	T*W	B	T*B	T*W*B
5th flushing	80.41 ± 2.33	77.44 ± 1.87	(−9.18; 3.23)	7.077	0.997	0.565	0.140	0.140	0.264	0.393
Estrus	87.98 ± 3.21	85.50 ± 3.82	(−12.90; 7.93)	5.320	0.388	0.436	0.197	0.515	0.127	0.150
N gilts	12	12								
	Triglycerides, mg/dL *									
	**Control**	**IPC**	**IC95%**	**RSD**	**T**	**W**	T*W	**B**	T*B	T*W*B
5th flushing	56.07 ± 5.89	58.40 ± 6.47	(−15.74; 20.40)	31.61	0.861	0.388	0.490	0.745	0.968	0.901
Estrus	74.54 ± 8.04	68.47 ± 6.12	(−27.20; 15.00)	21.81	0.995	0.435	0.672	0.632	0.855	0.487
N gilts	12	12								

IPC: Immunomodulatory protein concentrate. Treatment (T)-Control/IPC. Weight (W). Batches (B). Interactions between factors. T*W; T*B; T*W*B. * LS-means: Inter-estrus interval used as a covariate in the model (*p* < 0.05). RSD: Residual standard deviation.

## Data Availability

The original contributions presented in the study are included in the article; further inquiries can be directed to the corresponding authors.

## Abbreviations

The following abbreviations are used in this manuscript:

BFT	Backfat Thickness
BW	Bodyweight
IPC	Immunomodulatory Protein Concentrate
LD	Loin Depth
